# Peculiarities of mentalization that hamper consultations of patients with BPD

**DOI:** 10.1192/j.eurpsy.2022.1711

**Published:** 2022-09-01

**Authors:** E. Sokolova, A. Ryzhov

**Affiliations:** Lomonosov MSU, Faculty Of Psychology, Moscow, Russian Federation

**Keywords:** effectiveness of psychotherapy, cultural-historical model, mentalization, BPD

## Abstract

**Introduction:**

The uncertainty of COVID-pandemia, vital danger and disruptions in the habitual social contacts can be paralleled to the experiences of severe emotional stress and violence, usually found in the people with Borderline Personality Disorder. Both can be regarded as hampering the ability to categorize and express thoughts, feelings and experiences. The implementation of distant forms of psychological counseling may accentuate the mentalization deficiency.

**Objectives:**

To develop a theoretical framework for an empirical typology of impairments of mentalization.

**Methods:**

The model of consciousness proposed by L.S. Vygotsky was used for theoretical generalization of the levels of categorical structures of mentalization observed in previous empirical studies.

**Results:**

The following structures were identified: (1) the syncretic type of mentalization with low differentiation and complexity of object representations, their negative affective tone, autistic, chaotically mutable motivation and low emotional investment in relationships were described in patients with schizotypal disorders; (2) the “complex” type, with literal, non-generalized, field-dependent and rigid, or unstable, representation of the self, others and relationships as a result of the “fusion” of cognitive representations with the current emotional states. Similar types of mentalization were previously described in people with BPD and self-harming behavior (Sokolova, Laisheva, 2017).

**Conclusions:**

The ’syncretic’ and ’complex’ types of mentalization produce affective-cognitive distortions of the image of a psychotherapist, hamper the understanding of the conditional and metaphorical character of the therapeutic process, render difficult the de-traumatization of the unbearable experiences, and lessen the effectiveness of consultations of people with BPD.

**Disclosure:**

No significant relationships.

